# PEG Grafted Polymethacrylates Bearing Antioxidants as a New Class of Polymer Conjugates for Application in Cosmetology

**DOI:** 10.3390/ma13163455

**Published:** 2020-08-05

**Authors:** Justyna Odrobińska, Dorota Neugebauer

**Affiliations:** Department of Physical Chemistry and Technology of Polymers, Faculty of Chemistry, Silesian University of Technology, 44-100 Gliwice, Poland; justyna.odrobinska@polsl.pl

**Keywords:** antioxidants, conjugates, “click” chemistry, delivery systems, cosmetology

## Abstract

The amphiphilic copolymers of poly(ethylene glycol) methyl ether methacrylate (MPEGMA) and alkyne functionalized 2-hydroxyethyl methacrylate (AlHEMA) were synthesized by controlled atom transfer radical polymerization (ATRP). The reactions were carried out using the standard ATRP initiator ethyl α-bromoisobutyrate, (EiBBr) and the “bio”initiator bromoester derivative of 4-*n*-butylresorcinol (4nBREBr_2_). Two substances with antioxidant activity used in cosmetology, (±)-α-lipoic acid (LA) and ferulic acid (FA), were subjected to esterification reactions to introduce azide groups. The “click” reactions between the alkyne contained copolymers and functionalized acids (LA-N_3_, FA-N_3_) were performed to obtain polymer-antioxidant conjugates (P((HEMA-*click*-FA)-*co*-MPEGMA) and P((HEMA-*click*-LA)-*co*-MPEGMA)). The conjugation was performed with an efficiency of 20–75%. In vitro experiments in a phosphate buffer saline (PBS) solution at neutral conditions demonstrated that the sufficient release was reached after 2.5 h for FA and 1 h for LA. The rapid release kinetics as well as the polymer carriers, which were applied to regulate the delivery of antioxidant substances, are beneficial in cosmetology.

## 1. Introduction

Conjugates as a form of polymer carriers covalently binding a biologically active substances, i.e., natural or synthetic compounds, which after introduction into the body cause a therapeutic effect, have several advantages including the reduction of drugs’ immunogenicity, better bioavailability, prolonged-release, and improved action of the substance in the body [1]. Depending on the type of conjugated substance, conjugates, i.e., carrier–drug [2], carrier–protein (peptide) [3], and carrier–DNA [4], are distinguished. For carrier–drug conjugates, the drug may be bonded to the polymer either directly or via a linker. Additionally, a solubility modification group and a tropic molecule responsible for recognizing target tissues may be attached to the carrier [5]. The first conjugate of this type has been reported for poly(*N*-(2-hydroxypropyl)methacrylamide) (PHPMA) with doxorubicin (DOX) [6] using galactosamine as the tropic molecule [7]. In subsequent years, PHPMA conjugated with paclitaxel (PTX) [8], camptothecin (CPT) [9], and poly(ethylene glycol) (PEG) with CPT [10] and poly(glutamic acid)-PTX [11] have been tested in clinical trials. Nowadays, numerous polymer–drug conjugates have been obtained to report sensitivity to external stimuli [12,13], biodegradability [14], antibacterial properties [15,16], applications in nuclear imagining [17], therapy for bone diseases [18], wound healing and tissue regeneration [19], and, most commonly, for cancer therapy [20,21]. Among the common strategies of conjugate synthesis, the azide−alkyne cycloaddition, as the “click” chemistry reaction, has received great interest, because of the numerous advantages, i.e., efficiency, insensitivity to oxygen and water, regio- and stereospecificity, as well as mild reaction conditions and easy isolation of the product [22]. It has been used for the preparation of reduction-responsive polymeric prodrugs, i.e., polyphosphoester-CPT [23] or PEG-methotrexate (MTX) [24].

The delivery via conjugates is not limited to drugs, but can be extended to substances such as those useful in medical cosmetology, usually vitamins, such as vitamin E [25,26,27], vitamin B12 [28], folic acid [29], vitamin C [30] and vitamin A [31,32]. In most of these studies, they have played the role of a co-delivery substances to assist the carrier in the efficient delivery of anti-cancer drugs indicating a desired cytotoxicity against cancer cells and higher effect of apoptosis than the free drug, diverse drug resistance and reduced side inflammation, or cell protection from oxidative stress at allowed doses. The substances with antioxidant activities such as ferulic acid (FA) and lipoic acid (LA) have been previously conjugated to chitosan [33,34,35,36], polypeptides [37], cyclodextrin [38] and PEG [39]. FA is a free radical inhibitor with low toxicity and anti-inflammatory and anti-cancer properties, whereas in cosmetic preparations it is applied as a photoprotective substance, which brightens and delays the skin aging process [40]. LA is mainly used as a natural antioxidant compound, which also has a positive effect in various types of cancer [41]. The antioxidant action mechanism of FA and LA is based on the inhibition of the formation of reactive oxygen species or nitrogen, neutralization of free radicals, binding transition metals (iron and copper), and lipid peroxidation prevention [40,42].

**Figure 1:**
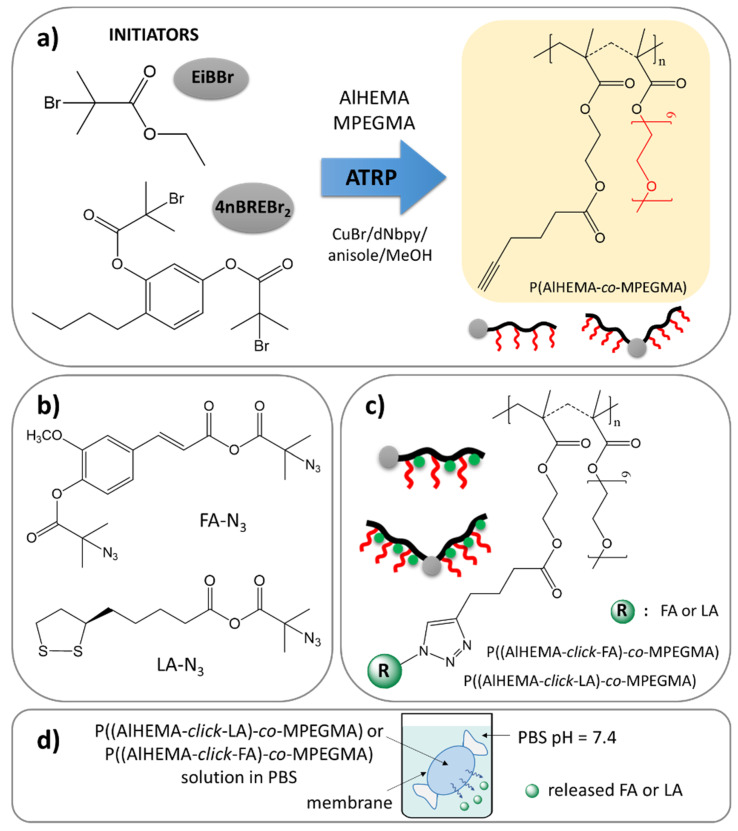
Synthetic route via copolymers obtained by ATRP (**a**), “click”-able antioxidants (**b**) to polymer-antioxidant conjugates (**c**), and their potential use as delivery systems (**d**).

Our earlier interest was devoted to the encapsulation of vitamin C and FA into the micelles of the self-assembling polymers [43] with an incorporated unit of vitamin A (retinol) [44] or its derivative (4-*n*-butylresorcinol) [45] as an accompanying biocompatible substance. Recently, we have focused on conjugate-type delivery systems with covalently attached FA and LA. The main part of the current study is to design amphiphilic graft copolymers of poly(ethylene glycol) methyl ether methacrylate (MPEGMA) and alkyne functionalized 2-hydroxyethyl methacrylate (AlHEMA) (P(AlHEMA-*co*-MPEGMA)), which can conjugate selected active substance (Figure 1). Additionally, the use of two different initiators in the copolymerization (monofunctional ethyl α-bromoisobutyrate (EiBBr) and bifunctional 4-*n*-butylresorcinol (4nBREBr_2_)) influences the topology of resultant copolymers, i.e., graft and V-shaped graft. The relatively high yield conjugation was accomplished using a “click” chemistry reaction between alkyne and azide groups as a convenient method to introduce LA or FA through their azide derivatives. The obtained polymer–antioxidant conjugates were physicochemically characterized, whereas their delivery capabilities were verified by monitoring of the in vitro release process under conditions similar to those on the human skin (PBS, pH = 7.4, 37 °C). The impact of the copolymer composition (relative ratios of MPEGMA/AlHEMA) and topology on the effectiveness of antioxidant conjugation seems to be crucial for future application of the designed conjugates as a beneficial delivery system in cosmetology in respect of the release rate and amount of active substance in the expected time interval.

## 2. Materials and Methods

### 2.1. Materials

Poly(ethylene glycol) methyl ether methacrylate (MPEGMA, Aldrich, M_n_ = 500 g/mol, 97%, Poznań, Poland), 2-hydroxyethyl methacrylate (HEMA, Aldrich, 97%, Poznań, Poland), methanol (Alfa Aesar, 99%, Warsaw, Poland) and anisole (Alfa Aesar, 99%, Warsaw, Poland) and copper (I) bromide (CuBr, Fluka, 98%, Steinheim, Germany) were prepared as was reported earlier [45]. 4,4-Dinonyl-2,2-dipyridyl (dNdpy, Aldrich, 97%, Poznań, Poland), *N*,*N*,*N*′,*N*″,*N*″-pentamethyldiethylenetriamine (PMDETA, Aldrich, 98%, Poznań, Poland), triethylamine (TEA, Aldrich, 99%, Poznań, Poland), pyridine (Aldrich, 99%, Poznań, Poland), 2-bromoisobutyryl bromide (BriBuBr, Aldrich, 98%, Poznań, Poland), 5-hexynoic acid (HexA, Acros, 97%, Geel, Belgium), 4-*n*-butylbenzene-1,3-diol (4nBRE, Ark Pharm, 95%, Gdańsk, Poland), ethyl α-bromoisobutyrate (EiBBr, Aldrich, 98%, Poznań, Poland), sodium azide (NaN_3_, Acros, 99%, Karlsruhe, Germany), *N*,*N*′-dicyclohexylcarbodiimide (DCC, Acros, 99%, Geel, Belgium), 4-dimethylaminopyridin (DMAP, Acros, 99%, Geel, Belgium), *N*,*N*-dimethylformamide (DMF, 99%, Chempure, Piekary Śląskie, Poland), tetrahydrofuran (THF, Chempure, Piekary Śląskie, Poland), 4-hydroxy-3-methoxycinnamic acid called as ferulic acid (FA, Acros, 99%, Geel, Belgium), (±)-α-lipoic acid (LA, Aldrich, 99%, Poznań, Poland), and 0.1 M sodium phosphate buffer saline (PBS; pH = 7.4, Aldrich, Poznań, Poland) were used as received.

### 2.2. Characterization

^1^H- and ^13^C- nuclear magnetic resonance (NMR) spectra were registered by means of a UNITY/INOVA spectrometer (300 MHz, Varian, Mulgrave, Victoria, Australia) for samples dissolved in dimethyl sulfoxide (DMSO), chloroform (CDCl_3_) or CDCl_3_ with a small addition of methanol (MeOD), whereas tetramethylsilane (TMS, 0 ppm) was an internal standard. Gas chromatography (GC, Agilent Technologies 6850 Network GC System, Santa Clara, CA, USA) with a flame ionization detector was used to determine conversions of monomers. The samples were dissolved in acetone, and then measured with optimization of the injector and detector temperature to 250 °C, the temperature of the column was increased from 40 °C to 200 °C. The signals of MPEGMA and AlHEMA at retention times 1.8 min and 10.0 min, respectively, were integrated in relation to anisole signal at 4.9 min. Gel permeation chromatography (GPC) system with an 1100 Agilent isocratic pump, degasser, thermostated columns, and differential refractometer MDS RI Detector was applied to determine the average molecular weights (M_n_) and dispersity indices (Đ). The samples were dissolved in tetrahydrofuran (THF) and the measurements were performed with a flow rate of 0.8 mL/min at 30 °C. Linear polystyrene with molecular weights ranged in 580–300,000 g/mol was used as a standard for calibration. Fourier-transform infrared (FT-IR) spectra were recorded at resolution of 4 cm^−1^ in a range of 4000–400 cm^−1^ (16 scans) by a Perkin–Elmer Spectrum Two 1000 FT-IR Infrared Spectrometer with option of attenuated total reflection (ATR) (Perkin Elmer, Waltham, MA, USA). Dynamic light scattering (DLS, Zetasizer Nano-S90, Malvern Technologies, Malvern, UK) with a He-Ne laser at a fixed scattering angle (173°) was applied to measure hydrodynamic diameters (D_h_) and polydispersity indices (PDI) of the polymer particles in aqueous solution at 25 °C. Ultraviolet-visible light spectroscopy (UV–Vis, Thermo Fisher Scientific Evolution 300, Waltham, MA, USA) was used to define the amount of contained antioxidant and monitor its release in PBS environment.

### 2.3. P(AlHEMA-co-MPEGMA) Synthesized in the Presence of EiBBr (Example for I)

dNdpy (41.05 mg, 0.101 mmol), MPEGMA (6.20 mL, 13.39 mmol), AlHEMA (1.00 g, 4.46 mmol), and solvent mixture (10 vol.% of monomers; MeOH: anisole = 1:9; methanol (0.072 mL) and anisole (0.648 mL)) were placed in a Schlenk flask and degassed by freezing in liquid nitrogen. Then, after addition of EiBBr (6.62 μL, 0.045 mmol) and re-degassing, the catalyst CuBr (6.40 mg, 0.045 mmol) was introduced. The reaction was carried out at 60 °C and the system was deactivated by contact with air, after observing a large increase in the density of the reaction mixture. To remove copper catalyst, the diluted in chloroform solution was passed through the column filled in a neutral alumina. The precipitation of polymer was performed in diethyl ether from the evaporated solution, and then it was dried under a vacuum.

### 2.4. P(AlHEMA-co-MPEGMA) Synthesized in the Presence of 4nBREBr_2_ (Example for III)

4nBREBr_2_ (20.63 mg, 0.045 mmol), dNdpy (41.05 mg, 0.101 mmol), MPEGMA (6.20 mL, 13.39 mmol), AlHEMA (1.00 g, 4.46 mmol), and solvents (10 vol.% of monomers; MeOH: anisole = 1:9; methanol (0.072 mL) and anisole (0.648 mL)) were placed in a Schlenk flask. After degassing, CuBr (6.46 mg, 0.045 mmol) was added to start reaction, which was carried out at 60 °C. The stop and purification steps were accomplished as it was presented in Section 2.3.

### 2.5. Modification of FA to the Azide Derivative (FA-N_3_)

FA (4.00 g, 20.62 mmol) was dissolved in 120 mL of anhydrous DMF yielding a colorless solution. TEA (5.80 mL, 41.24 mmol) was added and the flask content was cooled to 0 °C in an ice/water bath. After this, BriBuBr (5.10 mL, 41.24 mmol) was added gradually over 5 min. The reaction was completed within 24 h at room temperature and stirring in darkness. The mixture after transferring to a separator with chloroform was extracted consecutively with water (2 × 110 mL) and saturated NaCl solution (2 × 110 mL). The organic phase was filtered off and evaporated. The product (FA-Br) after drying under a vacuum was obtained with a yield of 81%.

FA-Br (10.78 g, 31.52 mmol) and NaN_3_ (2.23 g, 34.31 mmol) was dissolved in 50 mL of anhydrous DMF. The reaction at room temperature and without light was completed after 24 h. The further steps were analogous to the above-described procedure. The product (FA-N_3_) was obtained with a yield of 72%.

### 2.6. Modification of LA to the Azide Derivative (LA-N_3_)

TEA (2.00 mL, 14.35 mmol) was injected to a yellow solution of LA (2.45 g,11.89 mmol) dissolved in 60 mL of methylene chloride. The mixture was cooled to 0 °C, and then BriBuBr (1.78 mL, 14.40 mmol) was added gradually over 5 min. The further steps were analogous to the procedure presented in Section 2.5. The dried LA-Br was obtained with a yield of 86%.

LA-Br (3.02 g, 8.53 mmol) and NaN_3_ (0.60 g, 9.23 mmol) was dissolved in 50 mL of anhydrous DMF. The reaction was carried out for 24 h at room temperature and without light. The further procedure was analogous to that presented in Section 2.5. The dried LA-N_3_ was obtained with a yield of 81%.

### 2.7. CuAAC Reaction (Example for I-FA)

FA-N_3_ (0.07 g, 0.23 mmol) and PMDETA (0.12 mL, 0.58 mmol) were added to a solution of polymer I (0.40 g, 3.902 × 10^−3^ mmol with 0.23 mmol of AlHEMA units) in DMF (10 mL) and THF (10 mL). The reaction mixture after inert gas purge for 20 min was supplemented with CuBr (81.20 mg, 0.57 mmol) to start the reaction. After 48 h at room temperature and in darkness the reaction was finished. Purification from the catalyst was provided by cationite (Dowex), and precipitation of the product in diethyl ether.

### 2.8. Determination of Antioxidant Amount in Polymer

A solution of conjugate in PBS (pH = 7.4, 1.0 mg/mL) was analyzed by UV-Vis method to calculate the amount of attached antioxidant measuring the absorbance at λ = 310 nm for FA (ε = 824.3 cm^2^/mol, Figure S1) or λ = 334 nm for LA (ε = 6.6 cm^2^/mol, Figure S2) and correlating with a calibration curve. Additionally, the amount of conjugated active substance was determined by ^1^H NMR by calculating the number of triazole rings formed as a result of the “click” reaction.

### 2.9. Antioxidant Release

A solution of polymer conjugate in PBS (pH = 7.4, 1.0 mg/mL) was dialyzed using the cellulose membrane bag (MWCO = 3.5 kDa) in a vial with PBS (50 mL). The process was provided in a water bath at 37 °C. The samples of released medium were analyzed using UV–Vis method to measure the absorbance of FA or LA at proper wavelengths, and then the concentration of the released antioxidant was calculated.

## 3. Results

Two series of copolymers (I-V) were synthesized by atom transfer radical polymerization (ATRP) with a standard initiator EiBBr, as well as the bromoester functionalized 4nBRE (4nBREBr_2_) in the presence of the catalyst complex CuBr/dNbpy in anisole/methanol at 60 °C. The comonomer pair (AlHEMA/MPEGMA) with varying initial proportions (25/75, 75/25) was applied to control the hydrophilicity of polymers and content of alkyne groups crucial for the “click” reaction with azide sites. The latter ones were introduced in a two-stage modification of FA and LA, including esterification and azidation. The conjugation of the azide derivatives of selected antioxidants to the polymer matrix was completed via a Cu(I) catalyzed 1,3-dipolar azide-alkyne cycloaddition (CuAAC). This multi-step procedure is schematically presented in Figure 1.

**Figure 2:**
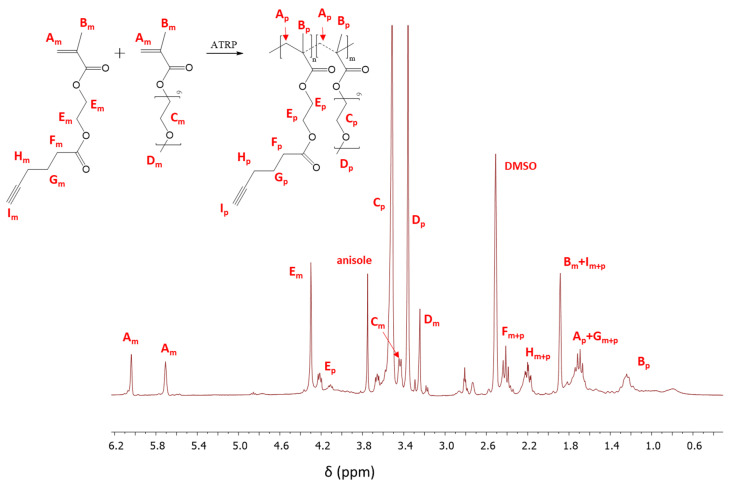
^1^H Nuclear Magnetic Resonance (NMR) spectrum of the reaction mixture for copolymerization of 2-hydroxyethyl methacrylate (AlHEMA)/amphiphilic graft copolymers of poly(ethylene glycol) methyl ether methacrylate (MPEGMA): 75/25 (II), m and p indicate the resonances that are related to monomer and polymer, respectively.

The structure of copolymers (I-V, Figure 1a) is confirmed by ^1^H NMR and FT-IR analysis. The resonances in the ^1^H NMR spectrum represent the protons of -CH_3_ groups in the main chain of the copolymer (B_p_: 0.6–1.5 ppm), those of the -OCH_2_CH_2_O- groups in side chains of MPEGMA units (C_p_: 3.51 ppm) and for the -COOCH_2_CH_2_OCO- groups in AlHEMA units (E_p_: 3.86–4.24 ppm) (Figure 2). The most characteristic groups in the obtained copolymers were also identified by FT-IR analysis. The peaks in the range of 550–700 cm^−1^ and 3300 cm^−1^ indicate the presence of alkyne groups, which causes bending and stretching vibrations of the ≡C–H, respectively (Figure S3). An increase in the intensity of both peaks was observed in the case where AlHEMA units are prevalent in the copolymer (Figure S3b). Similarly, the peak from the stretching vibrations of C=O (1720 cm^−1^) corresponds to ester groups in all repeating units, wherein each AlHEMA unit contains two ester groups in contrast to MPEGMA one. The opposite situation is observed in the band range from 1040 to 1200 cm^−1^, where stretching vibrations of C-O-C were mainly intensified by the MPEGMA units (Figure S3a).

The monomer conversion was determined from the ^1^H NMR spectrum through the integration of the resonances corresponding to the monomer and polymer. The conversion of AlHEMA was evaluated by the protons of the methylene groups in AlHEMA units (E_m_: 4.30 ppm; E_p_: 3.86–4.24 ppm). In the case of MPEGMA conversion, the peaks assigned to–OCH_2_CH_2_O–in the monomer and those in the polymer (C_m_: 3.45 ppm; C_p_: 3.51 ppm) were used for calculation (Figure 2). The conversion of the monomers was additionally determined by GC analysis which showed very good agreement with that obtained by ^1^H NMR, but the GC-based values were used to minimize the error of calculations of polymerization degree and molecular weight (DP, M_n,GC_).

The polymerization reactions were completed with monomer conversions in the range of 40–71% for AlHEMA and 24–60% for MPEGMA to obtain, in most cases, a DP above 160 (Table 1). Reaction IV, which took place over 5 h, resulted in an increase in conversion compared to the reaction III, yielding a polymer with a longer main chain (DP = 183 vs. 114, respectively). The initiator type, including the previously described “bio”initiator in the copolymerization of HEMA and methyl methacrylate or MPEGMA [45], is not a significant factor, which affects the rate of polyreaction with predominated AlHEMA (II, V), providing conversions of ~45%. In the case of systems with predominated MPEGMA, the initiation by the “bio”initiator resulted in a lower conversions when compared to the standard initiator (III vs. I), but they are still advantageous due to the improvement of skin treatment from the point of view of application in cosmetology. By comparing the relative comonomer conversions of AlHEMA vs. MPEGMA within the system, the statistical copolymers were obtained in the presence of a monofunctional initiator, i.e., EiBBr (I-II, X_AlHEMA_ ~ X_MPEGMA_), while in the case of the bifunctional initiator 4nBREBr_2_ the V-shaped copolymers with gradient were formed at twice lower conversion of MPEGMA than for AlHEMA.

**Figure 3:**
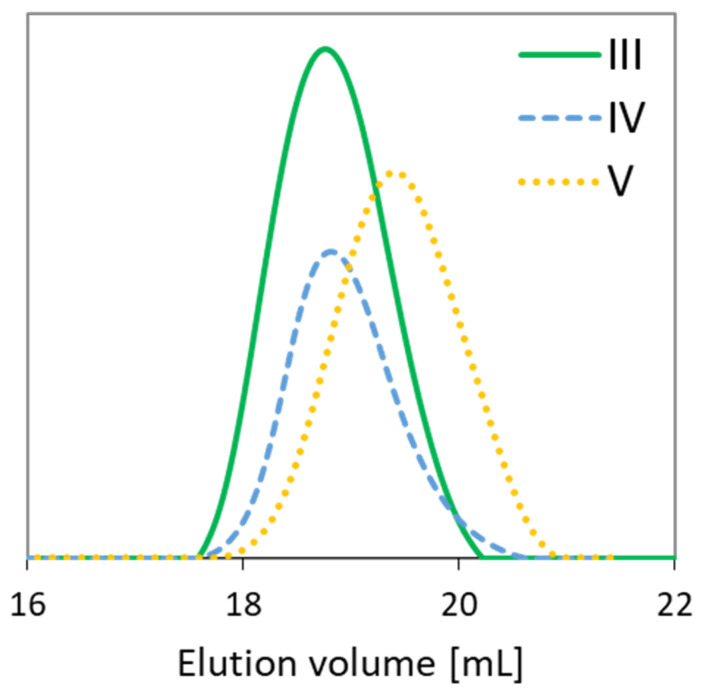
Gel permeation chromatography (GPC) traces of representative copolymers.

The relatively low dispersity indices (1.28–1.36) confirm the narrow molecular weight distributions due to the well-controlled polymerization processes (Table 1). The functionality of the initiators, i.e., EBiBr vs. 4nBREBr_2_, had no effect on the dispersity of polymers, but it is highly probable that a number of initiating groups (1 vs. 2) was crucial for the formation of two types of copolymers, grafted and V-shape grafted. The latter structure can be expected due to the presence of aromatic ring in the center of 4nBREBr_2_ and butyl substituent, which may cause steric hindrance, and limit rotation of the bromoester group especially in ortho position. The GPC traces were monomodal, but in some cases were nonsymmetrical (Figure 3). The reason for this may be steric hindrance caused by the specific structure of the monomers, i.e., AlHEMA with a substituent derived from hexic acid and MPEGMA with nine repeating methylene oxide units in the side chain. The discrepancy between the M_n_ values calculated from conversion analysis by GC and those determined by GPC analysis is due to the grafted topology of the copolymers, which have lower hydrodynamic volume in solution than the linear polymer standards used in the calibration of GPC. Moreover, the largest M_n_ divergence (M_n,GC_/M_n,GPC_ ~ 3) is observed for samples II and V with the lowest number of PEG grafts (DG < 30%) and the predominance of the hydrophobic fraction of AlHEMA (F_AlHEMA_ > 70%). The more hydrophobic nature of the polymer is beneficial for strong interactions with the column providing a longer elution time and allowing it to stay on the column, and this corresponds to a bigger divergence of M_n,GPC_ vs. M_n,GC_ than for the polymers I, III, IV with predominant MPEGMA and above 60% of DG (M_n,GC_/M_n,GPC_ > 2).

The azide derivatives of FA and LA (FA-N_3_, LA-N_3_, Figure 1b) were obtained using a two-step modification process. In the first stage, an esterification reaction was completed with BriBuBr and then the bromine atom was converted with a substitution reaction to an azide group. The formation of the bromoester derivatives (LA-Br, FA-Br) was observed by ^1^H NMR, which detects the appearance of a methyl group (H_a_) resonance at 1.96 ppm for LA modification (Figure S4b) and two equivalent signals from the methyl groups (H_h_: 1.94 ppm, H_a_: 2.08 ppm) in the case of FA modification (Figure S5b). The presence of two signals derived from methyl protons of the bromoisobutyrate groups in FA-Br can be explained by the esterification of both the hydroxyl and carboxyl groups resulting in signals from the methyl groups adjacent to the ester bond (H_a_, Figure S5b), which are shifted compared to those of the methyl groups adjacent to the anhydride bond (H_h_, Figure S5b). After azidation, the NMR signals from the methyl protons in the isobutyrate groups (H_a_, Figure S4b and H_a_, H_h_ Figure S5b) are displaced towards lower chemical shifts (H_a_: 1.51 ppm, Figure S4c and H_a_/H_h_: 1.54/1.49, Figure S5c) due to the exchange of the bromine to an azide group. The equivalence of both signals from the methyl groups in FA-Br and FA-N_3_ indicates complete esterification of the hydroxyl and carboxyl groups in FA. The final products were also characterized by ^13^C NMR analysis, where a signal from the carbons of methyl groups (Figure S6b, S7b: C11 at 23 ppm), as well as a signal from the carbon directly connected to the azide group (Figure S6b: C10 at 67 ppm; Figure S7b: C12 at 68 ppm) were observed. Additionally, the confirmation of the LA-N_3_ structure is seen by the occurrence of two equal signals derived from the carbons from the carboxylic anhydride, which was formed (Figure S6b: C8 at 173 ppm, C9 at 172 ppm) and the lack of signal from the carbon in the carboxyl group (Figure S6a: C8 at 177 ppm). For FA-N_3_, the appearance of a signal from the carbon in the ester bond (Figure S7b: C13 at 162 ppm) and two signals from the carbon in an anhydride bond (Figure S7b: C14 at 165 ppm and C1 at 161 ppm) is particularly important to prove the esterification of OH and COOH groups in the FA. This fact is also evidenced by the disappearance of the COOH carbon signal (Figure S7a: C1 at 167 ppm) and the appearance of its counterpart from the newly formed anhydride bond (Figure S7b: C1 at 161 ppm), as well as the shift of the carbon signal originally adjacent to the OH group (Figure S7a: C7 at 146 ppm) towards lower chemical shift due to the newly formed adjacent ester bond (Figure S7b: C7 at 141 ppm).

**Figure 4:**
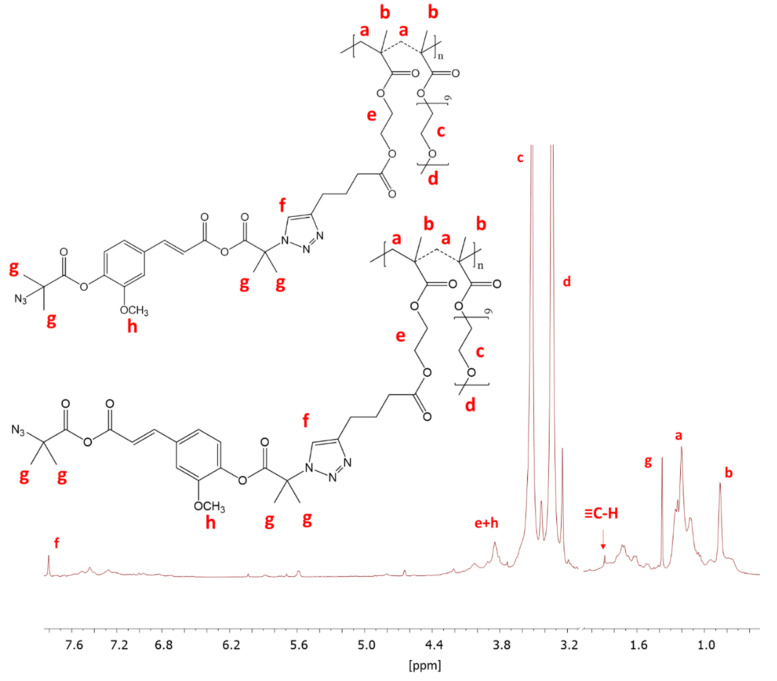
^1^H NMR of conjugated IV-FA.

The prepared FA-N_3_ or LA-N_3_ were conjugated to the P(AlHEMA-*co*-MPEGMA)s using a Huisgen “click” chemistry CuAAC reaction catalyzed by CuBr/PMDTA using azide and alkyne sites with the formation of 1,4-substituted triazole rings (Figure 1c). The presence of a triazole proton at 7.8 ppm (H_f_) in the ^1^H NMR spectrum validates the success of the “click” reaction (Figure 4). Because of the two azide groups in FA-N_3_, there are two possibilities to form a triazole ring, but the employment of both them in one FA molecule seems to be strongly limited by steric hindrance and shielding of alkyne sites by PEG grafts. The other intensive signals at 4.0 ppm (H_e_), 3.5 ppm (H_c_), 3.3 ppm (H_d_), 1.2 ppm (H_a_), 0.9 ppm (H_b_) correspond to protons in the polymer chain, whereas the signals at 3.8 ppm (H_h_) and, 1.4 ppm (H_g_) are representative for the protons in the conjugated FA units. The effectiveness of the “click” reaction (E_click_) was calculated using the H_f_ resonance and the signal of non-clicked alkyne groups at 1.9 ppm (H_≡CH_) using the following equation:

$$E_{\mathrm{click}}=\frac{H_{f}}{H_{f}+H_{\equiv \mathrm{CH}}}*100\%$$

Additionally, the E_click_ was determined with UV-Vis analysis, which resulted in similar values to those achieved through ^1^H NMR (maximum 1% difference), with three exceptions (Table 2). Because, in the case of ^1^H NMR analysis, some signals could be overlapping giving a large measurement error, the UV-Vis results were used for the further calculations regarding the amount of active substance released.

The “click” reactions with LA-N_3_ (II, IV) were carried out with high efficiencies (70–74%) yielding the highest molar drug content (DC = 38%). A larger content of the hydrophobic fraction in the polymer II matrix is advantageous to obtain a larger DC (F_AlHEMA_/DC: 73/38% vs. 39/27%), whereas there was no impact of the number of AlHEMA units on the reaction’s effectiveness (DP_AlHEMA_/E_click_: 119/74% vs. 71/70%). The “click” reactions with FA-N_3_ were performed with satisfactory efficiency for the copolymer I (I-FA, E_click_ = 52% at F_AlHEMA_ = 24%), but due to low content of alkyne moieties, a low amount of antioxidant was consequently attached (DC = 13%). Analogously, the high value of DC compared to that for II-LA was seen for the copolymer with the highest hydrophobic fraction content (V-FA, F_AlHEMA_ = 82%, DC = 32%). Comparing copolymers which have a similar content of hydrophobic fraction, but with different architecture (grafted II-FA vs. V-shape grafted V-FA), a strong correlation was evident showing twice the “click” effectiveness and a DC for the latter system (E_click_/DC = 17/13 vs. 39/32).

**Figure 5:**
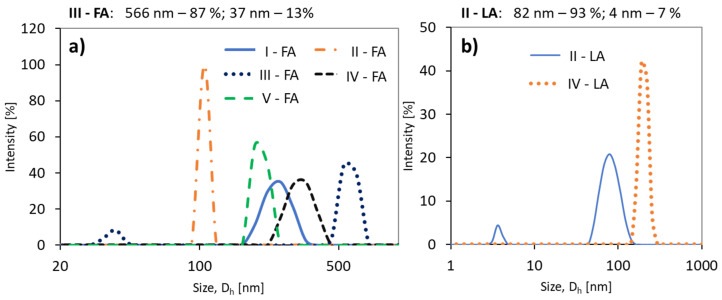
Size distribution intensity plots for (**a**) ferulic acid (FA) conjugates, and (**b**) (±)-α-lipoic acid (LA) conjugates.

The DLS analysis of the samples in PBS solution indicated the formation of homogeneous particles and monomodal signals except for the conjugates of copolymer II with LA and III with FA (Figure 5). Generally, both grafted and V-shape grafted conjugates show trends of reducing hydrodynamic diameters (D_h_) with an increase in the drug content (Table 2). A similar relationship has been previously reported for polymethacrylate–methotrexate conjugates [46]. By comparing the samples based on the same copolymer matrix (II or IV), to which LA and FA units were attached, it is noted that the particles of the LA conjugates are smaller (II-LA/II-FA = 82/106 nm or IV-LA/IV-FA = 202/323 nm) (Table 2).

**Figure 6:**
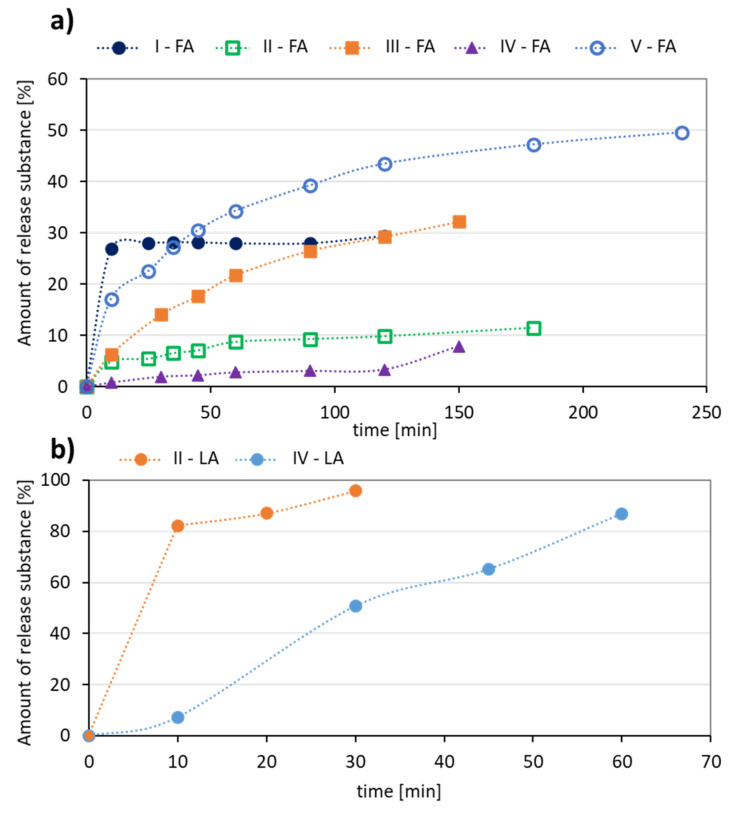
Kinetics profiles for (**a**) FA, and (**b**) LA release from the polymer conjugates.

The release experiments carried out in PBS at pH = 7.4 showed that this process occurred faster for LA systems yielding 87–96% of released antioxidant in up to 1 h (Figure 6b). Additionally, a higher content of the hydrophobic fraction in the conjugate promotes a large ejection of the substance immediately after the start of release and the maximum value was reached within half an hour (II-LA), while in the case of IV-LA, the release profile was gradual over 1 h (Figure 6b). The largest amount of FA (49%) was released from the conjugate V, with the highest DC and over the longest time (up to 4 h, Figure 6a). The other conjugates released 10–30% of FA as maximum amounts within 2–3 h.

**Figure 7:**
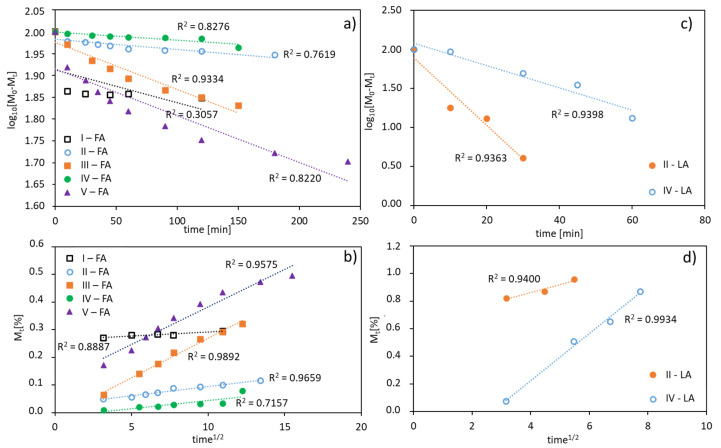
FA (**a**,**b**) and LA (**c**,**d**) release profiles from polymer conjugates fitted using a first-order equation (**a**,**c**), and a plot of the released drug vs. square root of time according to the Higuchi equation model (**b**,**d**).

The in vitro release profiles of both the antioxidants were fitted to kinetic models. The rate of FA or LA release is concentration dependent giving plots which fit well to first-order kinetics, expressed as a logarithm of the percentage of active substance remaining in the conjugate over time. The correlation coefficients (R^2^) was 0.94–0.95 for LA, 0.82–0.93 for FA released from V-shape grafted conjugates and 0.31–0.76 for FA released from the graft conjugates (Figure 7a,c). Moreover, the release data are plotted according to the Higuchi equation model, which describes the percentage of drug release as a function of the square root in time, to show a correlation of the system with diffusion process (Figure 7b,d). The plots for the studied conjugates show a good agreement with that model (R^2^ = 0.72–0.99), which confirms a concentration-dependent and diffusion-controlled mechanism. This means that the grafted topology of polymer and the self-assembling behavior of amphiphilic polymer in aqueous solution are crucial for diffusion process of released substance, which is slower than the hydrolysis of ester bonds connecting ferulic/lipoic units with the polymer matrix, and therefore the diffusion controls the release process.

## 4. Conclusions

The obtained amphiphilic copolymers of MPEGMA and AlHEMA with grafted topologies might differ in shape because of the used ATRP initiator, mono- or bifunctional (EiBBr or 4nBREBr_2_). The alkyne-functionalized copolymers were successfully modified using selective active substances with antioxidant properties (LA, FA) through the use of a “click” chemistry reaction with efficiencies ranging from 17–74% to design polymer–antioxidant conjugates. Generally, the “click” reactions showed that hydrophobic LA was effectively conjugated to a copolymer with a predominant content of hydrophobic fraction, while hydrophilic FA was more successful in the conjugation with a more hydrophilic copolymer, but a larger content of antioxidants was achieved for more hydrophobic copolymers. Most of the polymer conjugates provided particles with hydrodynamic diameters below 250 nm, the larger particles did not exclude these systems from potential application in cosmetology, as they can be used in the form of masks where a carrier which does not penetrate the skin can release the active substance. In vitro experiments performed in a PBS solution (pH 7.4) demonstrated that the maximum release of LA was after 0.5–1.0 h and for FA was after 2.0–4.0 h. This is beneficial for cosmetic products because they are applied for a short time in contact with the skin, and after that, the released substance can start the therapeutic process in deeper layers of the skin to achieve an optimal effect. The designed conjugates can be an alternative way for the delivery of antioxidants in cosmetology, where the polymer carrier can be responsible for controlled drug administration. The carriers with the best properties (high antioxidant content, a satisfactory amount of released substance in a relatively short time, and known release profile) have been selected for the evaluation of their biochemical potential, including cytotoxicity and penetration effect into the skin transdermal tests in the Franz chambers.

## Figures and Tables

**Table 1:** Data for AlHEMA/MPEGMA copolymers synthesized by ATRP.

M1/M2		Time (h)	Conversion (%)				DG ^a^ (%)	DP_n_^a^	M_n,GC_^a^ (g/mol)	M_n,GPC_^b^ (g/mol)	D ^b^
			X_NMR_		X_GC_						
			M1	M2	M1	M2					
I	25/75	2	59	58	58	60	76	238	102 500	54 400	1.29
II	75/25	2	44	44	40	45	28	163	49 000	18 500	1.35
III	25/75	2	54	17	41	24	64	114	45 800	28 300	1.35
IV	25/75	5	68	28	71	37	61	183	71 800	26 200	1.28
V	75/25	2	35	39	54	34	17	198	54 100	16 100	1.36

Conditions: [M1+M2]_0_/[I]_0_/[CuBr]_0_/[dNdpy]_0_ = 400/1/1/2.25, anisole/methanol = 9:1 10 vol% mon; 60 °C, where: M1 is AlHEMA and M2 is MPEGMA, I-II: EiBBr, III-V: 4nBREBr_2_^a^ calculated with the use of conversion by GC analysis, ^b^ determined by GPC in THF with polystyrene standards, DG–grafting degree.

**Table 2:** Data for obtained conjugates and release effect of active substances.

	DP_AlHEMA_	F_AlHEMA_ (mol.%)	E_click_^a^ (%)	E_click_^b^ (%)	n_tria._^a^	DC (mol.%)	D_h_ (nm)		PDI	R_AS_ (mol.%)	Time (h)
							Int.	Vol.			
I-FA	58	24	69	52	30	13	248 ± 36	250 ± 46	0.548	30	2.0
II-FA	119	73	18	17	20	13	106 ± 2	106 ± 11	1.000	12	3.0
II-LA	119	73	48	74	62	38	^c^ 82 ± 20	^c^ 71 ± 18	0.297	96	0.5
III-FA	41	36	38	38	16	14	^c^ 566 ± 46	^c^ 572 ± 75	1.000	32	2.5
IV-FA	71	39	34	33	23	20	323 ± 44	329 ± 57	0.836	8	2.5
IV-LA	71	39	57	70	50	27	202 ± 24	201 ± 32	0.694	87	1.0
V-FA	163	82	39	39	64	32	204 ± 15	204 ± 25	1.000	49	4.0

^a^ Determined by ^1^H NMR, ^b^ determined by UV-Vis; ^c^ value of particle size for dominated fraction; DP_AlHEMA_–polymerization degree of AlHEMA; F_AlHEMA_–content of AlHEMA in the copolymer; E_click_–efficiency of “click” reaction; n_tria._–number of triazole moieties in the copolymer; DC–drug content; R_AS_–released active substance.

## Supplementary Materials

The following are available online at https://www.mdpi.com/1996-1944/13/16/3455/s1, Synthesis Procedures: Modification of HEMA to form the alkyne derivative. Synthesis of bifunctional initiator. Figures S1 and S2: UV-vis absorption spectra of FA and LA. Figure S3: FT-IR spectra of copolymers of AlHEMA/MPEGMA. Figure S4: ^1^H NMR spectra of LA, LA-Br and LA-N_3_. Figure S5: ^1^H NMR spectra of FA, FA-Br and FA-N_3_. Figure S6: ^13^C NMR spectra of LA and LA-N_3_. Figure S7: ^13^C NMR spectra of FA and FA-N_3_.
