# Supplementary material for: PEG Grafted Polymethacrylates Bearing Antioxidants as a New Class of Polymer Conjugates for Application in Cosmetology
Source: Materials (Basel). 2020 Aug 5;13(16):3455. doi: 10.3390/ma13163455 (PMC7475907; doi:10.3390/ma13163455)
Supplement: Supplementary file 1 [file materials-13-03455-s001.pdf]

Synthesis Procedure:

Synthesis Procedure 1. Modification of HEMA to form the alkyne derivative (2-(prop-1-en-2-carbonyloxy)ethyl hex-5-ynate, AlHEMA)

Synthesis Procedure 2. Synthesis of bifunctional initiator (4-butyl-1,3-phenylene bis(2-bromo-2-methylpropanoate), $4nBREBr_2$)

Content:

Figure S1. UV-vis absorption spectra of FA.

Figure S2. UV-vis absorption spectra of LA.

Figure S3. FT-IR spectra of copolymers of AlHEMA/MPEGMA.

Figure S4. ^{1}H NMR spectra of LA, LA-Br and LA-N_3.

Figure S5. ^{1}H NMR spectra of FA, FA-Br and FA-N_3.

Figure S6. ^{13}C NMR spectra of LA and LA-N_3.

Figure S7. ^{13}C NMR spectra of FA and FA-N_3.

Synthesis Procedure 1. *Modification of HEMA to form the alkyne derivative (2-(prop-1-en-2-carbonyloxy)ethyl hex-5-ynate, AlHEMA)*

The AlHEMA monomer was obtained with a yield of 61% by esterification reaction with HexA, DCC and DMAP as we reported earlier [44]. ^{1}H-NMR (300 MHz, $CDCl_3$, ppm): 6.14 and 5.61 (2H, $=CH_2$), 4.35 (4H, $–OCH_2CH_2O–$), 2.52 (2H, $–OC(=O)CH_2–$), 2.28 (2H, $–CH_2$-C≡CH), 1.99 (1H, –C≡CH), 1.95 (3H, $–CH_3$), 1.81 (2H, $–OC(=O)CH_2CH_2–$). ^{13}C-NMR (75 MHz, DMSO, ppm): 172 (C7, $–OC(=O)CH_2–$), 166 (C4, –CC(=O)O), 136 (C2, CH_2=C–), 126 (C1, CH_2=C–), 83 (C11, –C≡CH), 72 (C12, –C≡CH), 63 (C5, $–OCH_2CH_2O–$), 62 (C6, $–OCH_2CH_2O–$), 32 (C8, $–OC(=O)CH_2–$), 27 (C9, $–OC(=O)CH_2CH_2–$), 18 (C10, $–CH_2$-C≡CH), 17 (C3, $–CH_3$). Electrospray ionization (ESI) MS (m/z): calculated for $C_{12}H_{16}O_4$, 224.0; found for $[M + Na]^+$, 247.1.

Synthesis procedure 2. *Synthesis of bifunctional initiator (4-butyl-1,3-phenylene bis(2-bromo-2-methylpropanoate), $4nBREBr_2$)*

The $4nBREBr_2$ "bio"initiator was synthesized with a yield of 97% by esterification reaction with BriBuBr and TEA according to a previously reported procedure [45]. ^{1}H NMR (300 MHz, DMSO, ppm): 7.18 (1H, –CH=, aromat.), 7.04 (1H, –CH=, aromat.), 7.02 (1H, –CH=, aromat.), 2.60 (2H, $–CH_2–$, aliphat.), 2.16 (12H, 2* $–C(CH_3)_2Br$), 1.59 (2H, $-CH_2-$, aliphat.), 1.38 (2H, $-CH_2-$, aliphat.), 0.98 (3H, $–CH_3$, aliphat.). ^{13}C NMR (75 MHz, DMSO, ppm) δ: 174 (C11, –OC(=O)–), 153 (C1, –CH=, aromat.), 149 (C3, –CH=, aromat.), 129 (C5, –CH=, aromat.), 128 (C4, –CH=, aromat.), 117 (C6, –CH=, aromat.), 114 (C2, –CH=, aromat.), 65 (C13, –OC(=O)**C**–), 42 (C7, C8, $–CH_2–$), 36 (C12, $–CH_3$), 29 (C9, $–CH_2–$), 18 (C10, $–CH_3$). ESI-MS (m/z): calculated for $C_{18}H_{24}Br_2O_4$ 462.0; found for $[M+Na]^+$ 486.0.

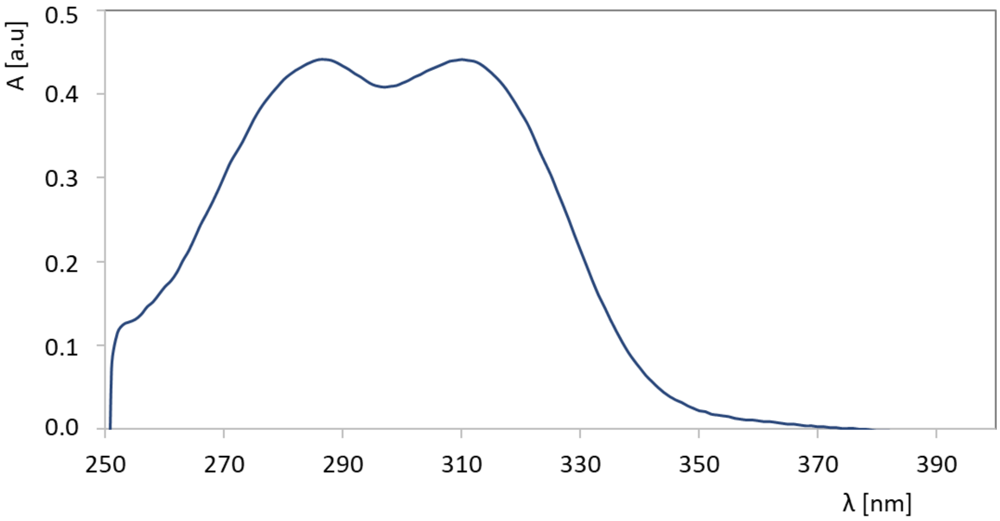

Figure S1. The absorption spectra of FA.

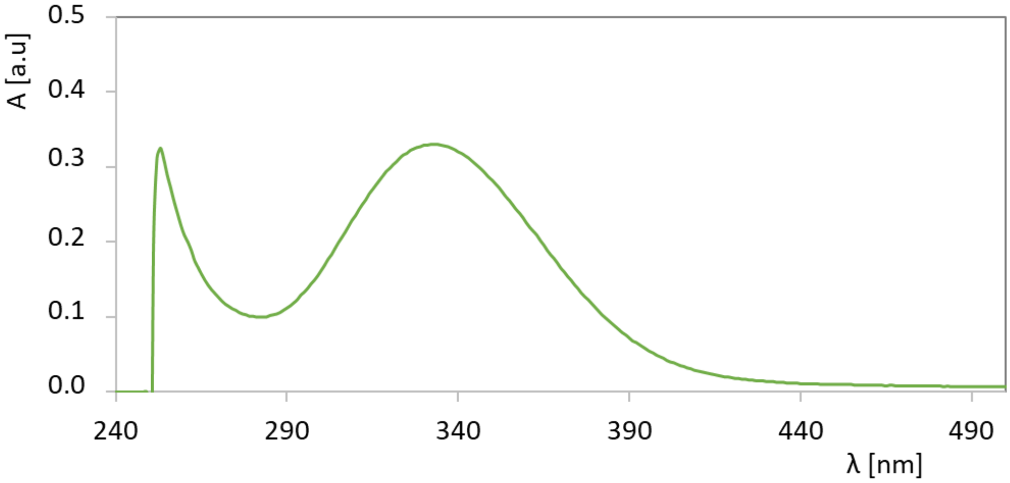

Figure S2. The absorption spectra of LA.

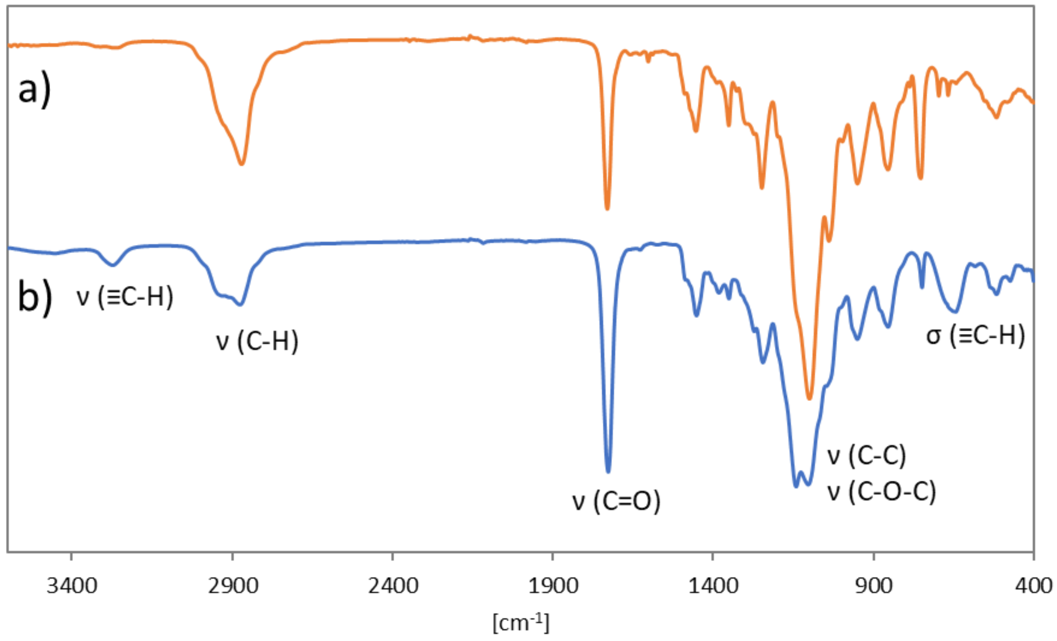

Figure S3. FT-IR spectra of copolymers of AlHEMA/MPEGMA: (**a**) 25/75 (III), (**b**) 75/25 (II).

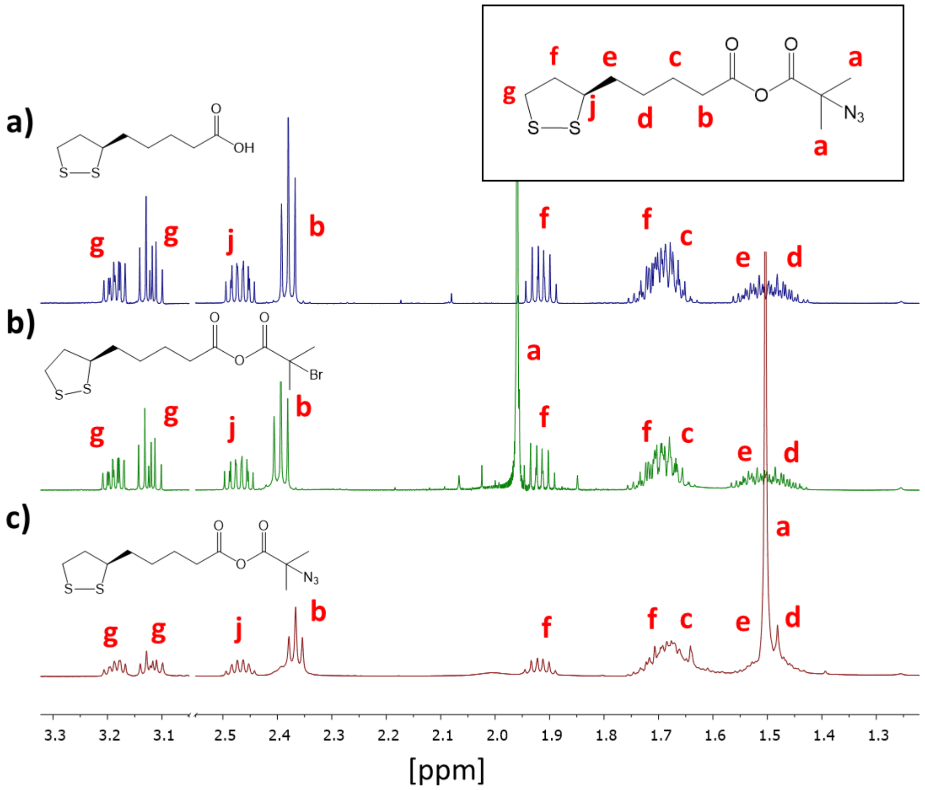

Figure S4. 1H NMR spectra of (**a**) LA, (**b**) LA-Br and (**c**) LA-N3.

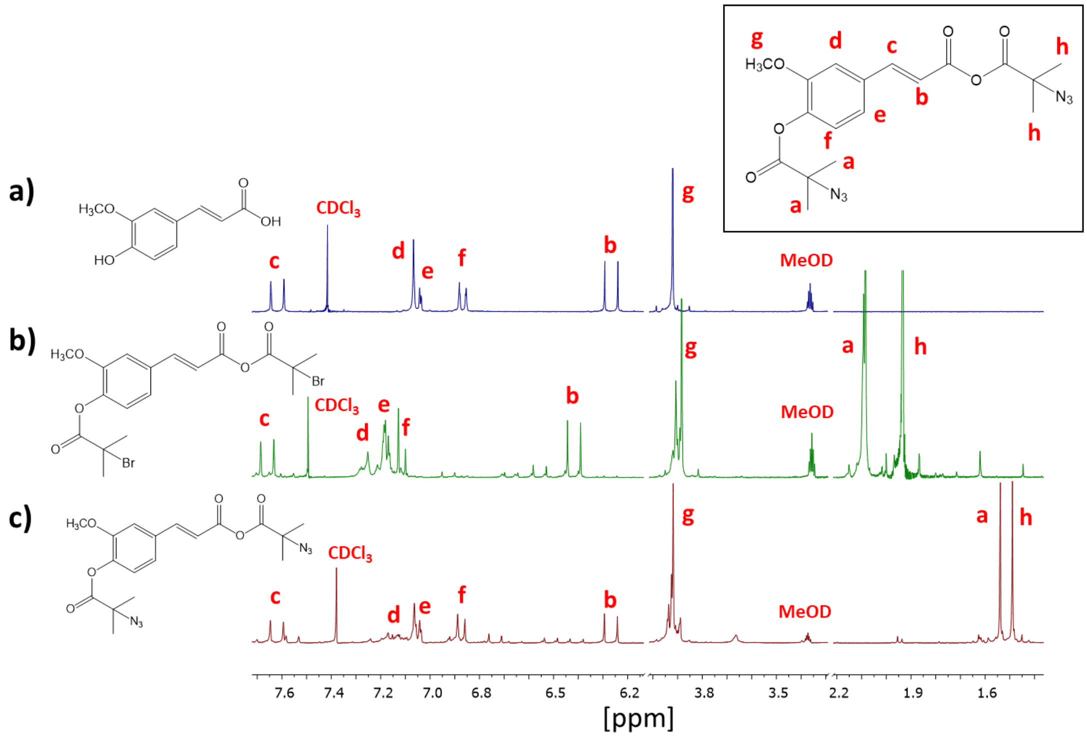

Figure S5. 1H NMR spectra of (**a**) FA, (**b**) FA-Br and (**c**) FA-N3.

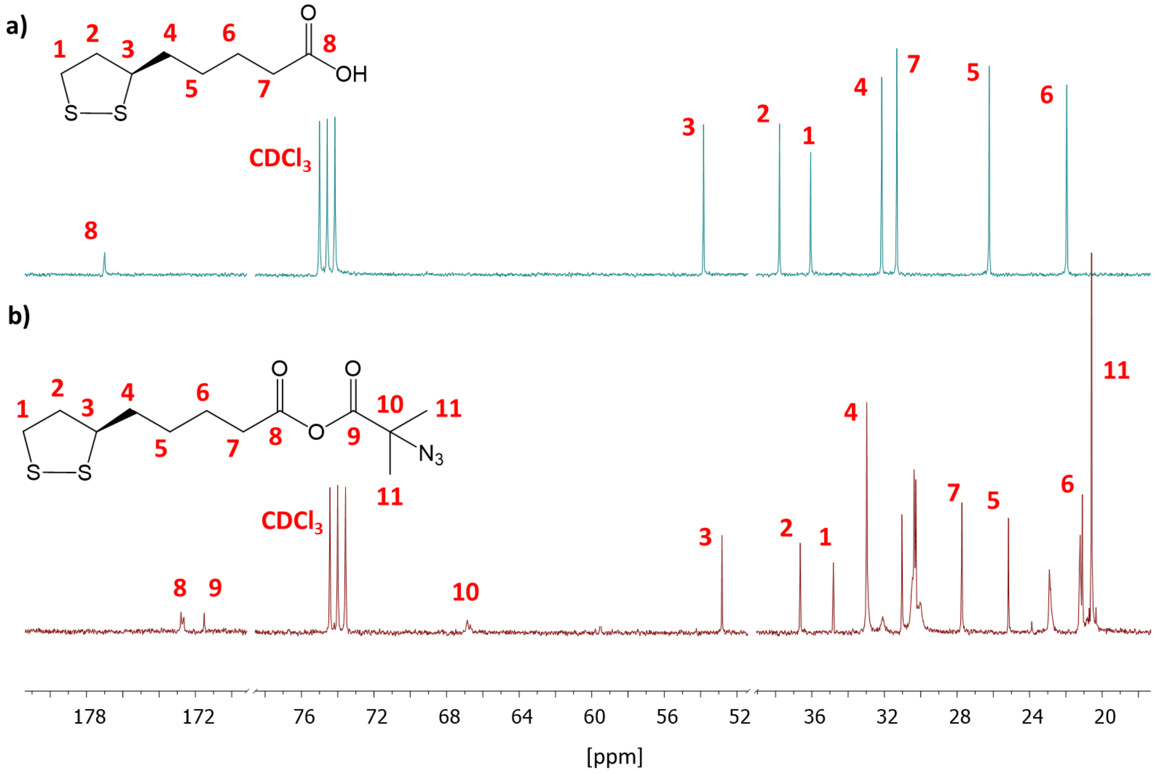

Figure S6. 13C NMR spectra of (**a**) LA, and (**b**) LA-N3.

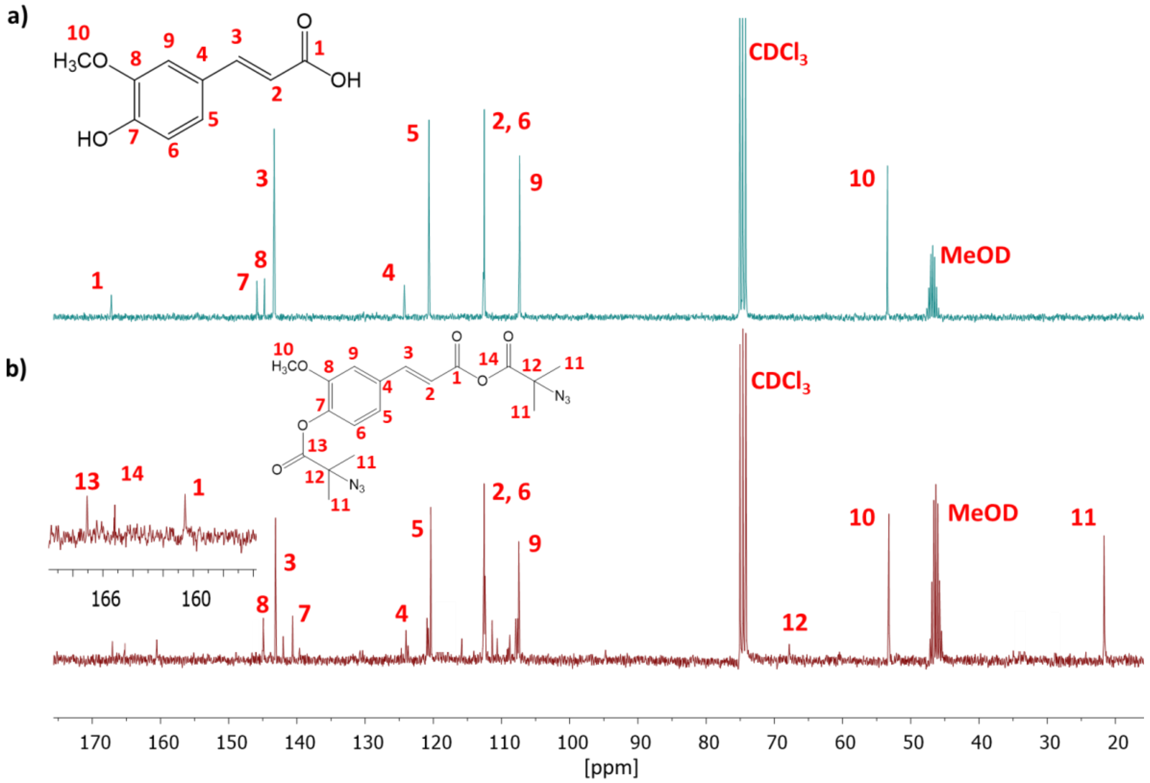

Figure S7. 13C NMR spectra of (**a**) FA, and (**b**) FA-N3.
